# Supplementary material for: Revision of the Lichen Genus Phaeophyscia and Allied Atranorin Absent Taxa (Physciaceae) in South Korea
Source: Microorganisms. 2019 Aug 6;7(8):242. doi: 10.3390/microorganisms7080242 (PMC6723189; doi:10.3390/microorganisms7080242)
Supplement: Supplementary file 1 [file microorganisms-07-00242-s001.zip › microorganisms-516652-List S1.pdf]

microorganisms

List S1. Examined specimens for the species of *Hyperphyscia*, *Phaeophyscia* and *Physciella* in Korea.

1. Hyperphyscia crocata Kashiw.

Examined specimens. JEOLLANAM PROVINCE. Yeosu City, Nam Town, Dumo road, Jickpo coast, Geumoh island, 34°43'10"N, 127°37'21"E, 6 m, on bark, 2012.04.26, S.Y. Kondratyuk 120413.

2. Phaeophyscia adiastola (Essl.) Essl.

Examined specimens. GANGWON PROVINCE. Hongcheon County, Nae Town, Mt. Eungbok, 37°51'47"N, 128°33'32"E, 866 m, on bark, 2009.05.23, Y. Joshi et al. 090741. Pyeongchang County, Daegwanryeong Town, Mt. Hwangbyeong, 37°44'41"N, 128°37'31"E, 630 m, on bark, 2008.07.14, J.S. Hur 080359, 080458, 080465. Samcheok City: Hajang Town, Mt. Sambong, 37°18'18"N, 128°56'20"E, 930 m, on bark, 2009.05.15, Y. Joshi et al. 090340, 090348; Singi Town, Macha road, Mt. Gitdaebong, 37°18'22"N, 128°56'48"E, 1222 m, on bark, 2009.05.15, Y. Joshi et al. 090415. Taebaek City, Mt. Taebaek, 1215 m, on bark, 2004.09.12, J.S. Hur 041040. Yangyang County, Seo Town, Galjeongokbong, 37°52'49"N, 128°31'2"E, 1020 m, on bark, 2009.05.22, Y. Joshi et al. 090533, 090234. Yeongwol County, Sangdong Village, Mt. Jang: 37°08'22"N, 128°51'5"E, 813 m, on bark, 2010.05.29, X.Y. Wang et al. 100886; 38°04'07"N, 128°27'03"E, 445 m, on bark, 2004.10.09, J.S. Hur 041304. GYEONGGI PROVINCE. Pocheon City, Sohol Village, Jikdong road, National arboretum, 37°45'14.05"N, 127°09'54.02"E, 222 m, on bark, 2014.07.10, R.U.G. Jayalal et al. 141209. GYEONGSANGNAM PROVINCE. Geochang County, Wicheon Town, Mt. Geumwon, 35°43'39"N, 127°45'50"E, 1351 m, on bark, 2010.06.25, X.Y. Wang et al. 100555. Hamyang County, Seosang Town, Mt. Baekun, 35°36'21"N, 127°39'22"E, 917 m, on bark, 2010.06.24, X.Y. Wang et al. 100380. Sancheong County, Sancheong Village, Mt. Ungseok, 35°22'53"N, 127°51'01"E, 0 m, on bark, 2007.10.16, J.S. Hur 070851. JEOLLABUK PROVINCE. Gochang County, Asan Town, Mt. Seonun, 36°57'27"N, 128°26'36"E, 2003.05.11, J.S. Hur 030246. JEOLLANAM PROVINCE. Gurye County, Toji Town, Mt. Jiri, 35°19'14"N, 127°39'46"E, 1491 m, on bark, 2006.09.29, J.S. Hur 060923.

3. Phaeophyscia endococcinodes (Poelt) Essl.

Examined specimens. CHUNGCHEONGNAM PROVINCE. Seosan City, Buseok Town, Buseoksa, 36°57'33"N, 128°29'18"E, 1364 m, on rock, 2003.10.04, J.S. Hur 030833. JEOLLANAM PROVINCE, Sinan County, Bigeum Town, Mt. Sunwang, Bigeum island, 34°44'14" N, 125°55'48" E, 92 m, on rock, 2013.06.05, S.O. Oh et al.130163.

4. Phaeophyscia exornatula (Zahlbr.) Kashiw.

Examined specimens. CHUNGCHEONGBUK PROVINCE. Danyang County, Gagok Town, Mt. Sobaek: 36°57'33"N, 128°29'18"E, 1375 m, on bark, 2003.10.02, J.S Hur 030763; 36°53'32"N, 128°26'06"E, 730 m, on bark, 2007.06.10, J.S Hur 070377, 070414; 36°32'39"N, 127°50'42"E, 575 m, on moss and rock, 2006.04.22, J.S Hur 060042. Goesan County, Yeonpung Town, Mt. Joryeong forest resort, 37°48'27"N, 128°03'32"E, 500 m, on *Quercus*, 2008.07.10, J.S Hur 080302, 080305; Sinseon Peak, 36°48'46"N, 128°02'44"E, 590 m, on *Quercus*, 2006.10.27, J.S Hur 061023, 061028. Sangchon Town, Mulhan road, Mt. Samdobong, 36°01'15"N, 127°52'41"E, 982 m, on trunk, 2015.07.01, S.O. Oh et al. 150435. Gongju City, Banpo Town, Mt. Gyeryong, 36°21'48"N, 127°13'15"E, 510 m, on rock, 2004.10.23, J.S. Hur 041618, 041622. GANGWON PROVINCE, Gangreung City, Jeongseon County, Okgye Town, Mt. Seokbyeong, Imgye Town, 37°34'29"N, 128°51'37"E, 774 m, on *Quercus*, 2008.05.24, J.S Hur 080202. Hoengseong County, Dunnae Town, Cheongtaesan, 1172 m, on bark, 2013.06.15, S. O. Oh & J. S. Park 130532. Hongcheon County, Nae Town, Mt. Eungbok, Tongbaram Valley, 37°51.359'N, 128°30.974'E, 1192 m, on moss over rock, 2009.05.23, Y. Joshi et al. 090672. Jeongseon County, Bukpyeong Town, Mt. Baekseokbong, 37°28.739′N, 128°39.760′E, 494 m, on moss over rock, 2009.05.16, Y. Joshi et al. 090425, 090459, 090492. Pyeongchang County: Daegwanryeong Town, Mt. Hwangbyeong,

37°44'41"N, 128°37'31"E, 630 m, on moss over rock, 2008.07.14, J.S Hur 080354, 080358, 080469; Yongpyeong Town, Mt. Gyebang, 37°42'48"N, 128°28'32"E, 772 m, on moss over rock, 2008.07.16, J.S Hur 080662. Samcheok City: Hajang Town, Mt. Sambong, 37°18.306'N, 128°56.368'E, 930 m, on *Quercus*, 2009.05.15, Y. Joshi et al. 090359; Singi Town, Macha road, Mt. Gitdaebong,, 37°18.367'N, 128°56.766'E, 1222 m, on rock, 2009.05.15, Y. Joshi et al. 090405: Wondeok Village, Mt. Keumbong, 37°14'10"N, 129°17'23 "E, 0 m, on bark, 2015.07.11, J. S. Park 151858-1. Sokcho City, Mt. Seorak, 38°10'23"N, 128°18'18"E, 355 m, on moss over rock, 2005.06.16, J.S Hur 050232; 38°09.981'N, 128°27.267'E, 463 m, on bark, 2009.05.24, Y. Joshi et al. 090753; on *Quercus*, 2009.05.24, Y. Joshi et al. 090774. Taebaek City, Mt. Taebaek, 37°06'28"N, 128°56'48"E, 944 m, on moss over rock, 2003.08.20, J.S Hur 030625. Wonju City, Socho Town, Mt. Chiak, 37°17'44"N, 128°01'20"E, 515 m, on rock, 2004.08.13, J.S. Hur 040569. Yangyang County, Mt. Seorak, Heulrimgol valley, 38°05.036'N, 128°25.176'E, 884 m, on *Carpinus*, 2009.05.25, Y. Joshi et al. 090930, 090933. Yangyang County: Seo Town, Galjeongokbong, 37°52.148'N, 128°30.881'E, 1087 m, on *Quercus*, 2009.05.22, Y. Joshi et al. 090645, 090211, 090240; Yangpyeong Village, Mt. Bongmisan, 37°36'11"N, 127°36'13"E, 191 m, on bark, 2008.07.31, J.S Hur 080341. GYEONGSANGBUK PROVINCE. Bonghwa County, Mulya Town, Mt. Seondal: 37°01'03"N, 128°43'19"E, 485 m, on moss over rock, 2007.04.24, J.S Hur 070216; 37°01'19"N, 128°42'08"E, 598 m, on bark, 2007.04.24, J.S Hur 070228. Cheongsong County, Budong Town, Mt. Juwang, 36°23'59"N, 129°09'53"E, 310 m, on moss and rock, 2005.10.16, J.S Hur 050621. Mungyeong City, Mungyeong Village, Mt. Juheul, 36˚46'30"N, 128˚06'13"E, on bark, 2004.02.29, J.S Hur 040133. Sancheong County, Sicheon Town, Mt. Jiri, Jungsan road, 35°19'10"N, 127°44'31"E, 1265 m, on moss and rock, 2006.09.15, J.S Hur 060657. GYEONGSANGNAM PROVINCE. Hamyang County, Macheon Town: Chang-won road, Mt. Baekun, 35°38'18"N, 127°37'27"E, 1087 m, on trunk, 2015.07.04, S.O. Oh et al. 150747; 35°36'17"N, 127°38'27"E, 848 m, on moss and rock, 2006.08.17, J.S Hur 060578; Mt. Jiri, 35°20'32"N, 127°41'09"E, 715 m, on moss over rock, 2004.09.04, J.S Hur 040923. Hapcheon County, Gaya Town, Mt. Gayasan, 36°48'34"N, 128°08'29"E, 540 m, on moss and rock, 2006.05.05, J.S Hur 060099, 060101, 060105, 060109. Namhae County, Changseun Island, 34°50'27" N, 127°58'41" E, 2 m, on rock, 2011.04.29, X.Y. Wang & J.A. Ryu, 110260, 110156, 110161. Sancheong County, Sicheon Town, Jungsan road, Mt. Jiri, 35°19'37"N, 127°44'16"E, 1479 m, on bark, 2006.09.15, J.S Hur 060683. Tongyeong City: Salyang island, 34°50.590'N, 128°12.137'E, 28 m, on rock, 2011.04.20, X.Y. Wang et al. 110026; Yokji Town, Seosan road: Duckdong beach, Yokji island, 34°38'03"N, 128°14'15"E, 1 m, on rock, 2012.05.11, S.O. Oh et al. 120744, 120789, 120790, 120791, 120830; Udong beach, Yokji island, 34°37'05"N, 128°14'38"E, 8 m, on rock, 2012.05.11, S.O. Oh et al. 120844. Yangsan City, Wondong Town, Mt. Cheontae, 36°09'15"N, 127°36'60"E, 234 m, on moss and rock, 2006.11.03, J.S Hur 061164. INCHEON METROPOLITAN CITY. Ganghwa County, Samsan Town, Seokmo island, 37°41.381'N, 126°19.320'E, 160 m, on moss over rock, 2010.09.29, X.Y. Wang et al. 101045. Ongjin County, Baengnyeong Town, Baengnyeong island, 12 m, on rock, 2013.06.12, S.O. Oh et al. 130407, 130499. JEJU PROVINCE. Jeju City: Chuja island, Chuja Town, Sinyang-1 road, seashore of Mojini-mongdol, 33°56'45"N, 126°20'03"E, 57 m, on rock, 2014.06.21, L. Lokos, 140836-2; Gujwa Village, Gimnyeong road, Gimnyeong port, 33°33'26"N, 126°43'57"E, on rock, 2004.08.29, J.S. Hur, 040890; Mt.Halla, Gwaneumsa Trail, 33°24'39"N, 126°32'47"E, 739 m, on tree, 2012.06.01, S.O. Oh et al. 121009; seaside, 33°33'54"N, 126°45'48"E, 10 m, on rock, 2009.04.19, J.S. Hur et al. 090029. Seogwipo City: Gangjeong, Yeongtto waterfall, 33°16'02"N, 126°29'49"E, 210 m, on rock, 2014.06.19, Y. Joshi 140579; Namwon Village, Wimi road, 33°16'13"N, 126°39'30"E, 10 m, on rock, 2009.05.29, J.S. Hu et al. 091414, 091423, 091433. Yongdam, 33°30'57"N, 126°30'38"E, 10 m, on rock, 2009.04.20, J.S. Hur et al. 090077, 090085. JEOLLABUK PROVINCE. Gunsam City, Shinsi island, 35°49'09"N, 126°27'56"E, 19 m, on rock, 2011.08.22, X.Y. Wang & J.A. Ryu, 110829. Jangsu County: Janggye Town, Odong road, Mt. Gusibong, 35°42'25"N, 127°39'23"E, 882 m, on *Quercus* sp., 2015.07.03, J.J. Woo et al. 150672; Jangsu Village, Daeseong road, Mt. Palgongsan, 35°36'19"N, 127°28'14"E, 873 m, on moss over rock, 2005.09.10, J.S Hur 050405, 050408. Jeongeup City, Mt. Naejang, 463 m, on rock, 2003.08.08, J.S. Hur 030601. Jinan County, Bugwi Town, Suhang road, Mt. Bugwisan, 35°48'00"N, 127°23'55"E, 645 m, on bark, 2005.09.11, J.S Hur 050425, 050426. Muju County: Mupung Town, Mt. Daedeok, 35°54'40"N, 127°53'13"E, 1156 m, on *Quercus*, 2015.06.19, J.J. Woo et al. 150156; Seolcheon Town, Mt. Deogyu,

35°51'03"N, 127°44'55"E, 1578 m, on moss over rock, 2005.04.30, J.S Hur 050183.Namwon City, Ayeong Town, Gusang road, Mt. Bonghwa, 35°31'52"N, 127°34'06"E, 685 m, on tree, 2015.07.17, S.O. Oh et al. 152193. JEOLLANAM PROVINCE. Gurye County, Masan Town, Mt. Jiri, Hwaeom valley, 35°16.905′N, 127°31.003′E, 816 m, on bark, 2009.10.12, Y. Joshi et al. 091045, 091055; 35°18'42"N, 127°35'59"E, 1485 m, on bark, 2006.09.28, J.S Hur 060837. Jindo County: Hachodo island, 34°19'05"N, 126°02'23"E, 3 m, on rock, 2011.08.23, X.Y. Wang & J.A. Ryu, 110879; Jeob island, 34°23'41" N, 126°18'9" E, 1 m, on rock, 2011.06.03, X.Y. Wang & J.A. Ryu, 110489. Shinan County: Anzua Island, 34°45'22"N, 126°7'29"E, 1 m, on rock, 2011.06.02, X.Y. Wang & J.A. Ryu, 110427; Bogil Island, 34°09.292'N, 126°34.721'E, 2 m, on rock, 2011.06.22, X.Y. Wang & J.A. Ryu, 110615; Sinui Town, Hatae road seaside, Sinuido, 11 m, on rock, 2013.06.28, S.O. Oh et al. 130540. Wando County: Bogil Town, Bogil island: Jung road, Jungri beach seaside, 34°09'47"N, 126°35'29"E, 13 m, on rock, 2010.02.06, Y. Joshi et al. 100215; Tong road, near Tongri beach, 34°09'30"N, 126°35'9"E, 15 m, on rock, 2010.02.06, Y. Joshi et al. 100163; Geumil Village, Wolsong road, Geumil island, 34°20'29"N, 127°02'38"E, 1 m, on rock, 2012.04.19, U. Jayalal et al. 120252, 120268. Yeonggwang County, seaside, 35°19'34"N, 126°22'42"E, 5 m, on rock, 2011.06.01, X.Y. Wang & J.A. Ryu 110318. Yeosu City: Hwayang Town, Imok road, Baelga coast, 34°39'00" N, 127°34'06" E, 12 m, on rock, 2012.04.28, U. Jayalal et al. 120646; Nam Town, Dumo road, Jickpo coast, Geumoh island: 34°30'45"N, 127°44'14"E, 6 m, on rock, 2012.04.26, U. Jayalal et al. 120358; 34°30'30"N, 127°46'13"E, 14 m, on rock, 2012.04.27, U. Jayalal et al. 120600; Samsan Town, Geomun island, 34°00'14"N, 127°19'29"E, 60 m, on rock, 2007.03.23, J.S. Hur, 070060, 070063; 1 m, on rock, 2007.03.24, J.S. Hur 070168.

5. Phaeophyscia hirtella Essl.

Examined specimens. CHUNGCHEONGBUK PROVINCE. Danyang County, Gagok Town, Mt. Sobaek, on bark, 2003.10.02, J.S. Hur 030738. Goesan County, Yeonpung Town, Mt. Joryeong forest resort, 37°48'27"N, 128°03'32"E, 500 m, on bark, 2008.07.10, J.S. Hur 080332. Jecheon City, Hansu Town, Mt. Worak, on Ginkgo, 2004.09.18, J.S. Hur 041167. GANGWON PROVINCE. Pyeongchang County: Yongpyeong Town, Mt. Gyebang, 37°42'42"N, 128°28'13"E, 1299 m, on *Quercus*, 2008.07.16, J.S. Hur 080658; Daegwanryeong Town, Mt. Hwangbyeong: 37°44'41"N, 128°37'31"E, 630 m, on rock, 2008.07.14, J.S. Hur 080357; 37°44'44"N, 128°37'30"E, 779 m, on *Quercus*, 2008.07.14, J.S. Hur 080366; Jinbu Town, Mt. Odae: 37°46'04"N, 128°36'12"E, 1277 m, on *Quercus*, 2008.07.15, J.S. Hur 080510; 37°46'22"N, 128°36'06"E, 1418 m, on bark, 2008.07.15, J.S. Hur 080596. Samcheok City, Singi Town, Macha road, Mt. Gitdaebong: 37°18'19"N, 128°56'43"'E, 1222 m, on bark, 2009.05.15, Y. Joshi et al. 090388; 37°18'19"N, 128°56'43"'E, 1222 m, on *Quercus*, 2009.05.15, Y. Joshi et al. 090412. Taebaek City, Sodo, Mt. Taebaek: on rock, 2003.08.20, J.S. Hur, 030632; 37°05'15"N, 128°57'17"E, 1070 m, on *Acer*, 2007.06.18, J.S. Hur 070561. Yangyang County, Seo Town, Hwangi road, Mt. Jobong, 37°56'6"N, 128°33'42"E, 980 m, on *Acer*, 2009.05.14, Y. Joshi et al. 090251. Yeongwol County, Sangdong Village, Gurae road, nearby Manhang-jae, 37°9'21"N, 128°53'28"E, 1260 m, on *bark*, 2014.07.17, U. Jayalal et al. 141333, 141337; Sangdong Village, Mt. Jang, 37°08.387'N, 128°51.042'E, 762 m, on bark, 2010.05.29, U. Jayalal et al. 100868. GYEONGGI PROVINCE. Gapyeong County, Buk Town, Mt. Myeongji, 37°55'58"N, 127°28'53"E, 223 m, on *Salix*, 2008.09.20, J.S. Hur 080666. Yangpyeong County, Yangpyeong Village, Mt. Bongmisan, 37°36'03"N, 127°35'14"E, 278 m, on *Castanea*, 2008.07.31, J.S. Hur 080342. GYEONGSANGBUK PROVINCE. Bonghwa County, Mulya Town, Mt. Seondal, 37°00'58"N, 128°43'21"E, 278 m, on bark, 2007.04.24, J.S. Hur 070211. INCHEON METROPOLITAN CITY. Ganghwa County, Hwado Town, 37°42'41"N, 126°23'27"E, 52 m, on *Zelkova*, 2010.09.30, 101132. JEJU PROVINCE. Jeju City, Arail, Sancheon-dan, 33°26'50"N, 126°33'15"E, 371 m, on bark, 2015.12.17, J.S. Park 152879. JEOLLABUK PROVINCE. Gochang County, Asan Town, Seonun Temple, 35°29'56"N, 126°35'17"E, 10 m, on bark, 2004.02.20, J.S. Hur 040046. Jangsu County, Janggye Town, Odong road, Mt. Gusibong, 35°42'59"N, 127°39'35"E, 767 m, on *Quercus*, 2015.07.03, J.J. Woo et al. 150607. Muju County, Seolcheon Town, Mt. Deogyu, 35°53'03"N, 127°46'46"E, 633 m, on *Koelreuteria*, 2005.04.02, J.S. Hur 050034. JEOLLANAM PROVINCE. Hwasun County, Doam Town, Mt. Cheonbul, Unju Temple, 34°55'13"N, 126°52'51"E, 70 m, on bark, 2005.09.04, J.S. Hur 050378.

6. Phaeophyscia hirtuosa (Kremp.) Essl.

Examined specimens. JEJU PROVINCE, Jeju City, 33°27'15"N, 126°33'41"E, 370 m, on bark, 2004.08.29, J.S. Hur 040859.

7. Phaeophyscia hispidula (Ach.) Essl.,

Examined specimens. GANGWON PROVINCE. Jeongseong County, Gangneung City, tourist pass toward peak Seokbyeongsan, 37°34'37"N, 128°51'47"E, 840 m, on bark, 2015.07.10, J.J. Woo et al. 151051. Sokcho City, Mt. Seorak, 38°09'58"N, 128°27'17"E, 463 m, on *Quercus*, 2009.05.24, Y. Joshi et al. 090817. Yangyang County, Mt. Seorak, Heulrimgol valley, 38°05'25"N, 128°24'39"E, 750 m, on bark, 2009.05.25, Y. Joshi et al. 090871. GYEONGSANGNAM PROVINCE. Hamyang County, Seosang Town, Mt. Baekun, 35°36'20"N, 127°39'38"E, 917 m, on *Quercus*, 2010.06.24, X.Y. Wang et al. 100400. JEOLLABUK PROVINCE. Muju County, Seolcheon Town, Mt. Sambong, 35°52'08"N, 127°49'42"E, 970 m, on *Quercus*, 2015.06.18, J.J. Woo et al. 150046. JEOLLANAM PROVINCE. Gwangyang City, Okryong Town, Mt. Baekun, 35°04'39"N, 127°39'38"E, 675 m, on *Quercus*, 2004.09.25, J.S. Hur 041254. Wando County, Gunoe Town, Wando Arboretum, 34°21'10"N, 126°41'11"E, 535 m, on rock, 2005.04.13, J.S. Hur 050151. Chuncheon City, Buksan Town, Jogyo road, Mt. Maebong, 37°54'38"N, 127°58'54"E, 610 m, on bark, 2010.05.26, X.Y. Wang et al. 100569.

8. *Phaeophyscia hunana* G.R. Hu & J.B. Chen

Examined specimens. JEJU PROVINCE, Jeju City, Halla Mt., Seongpanak Trail, 33°22'48"N, 126°35'27"E, 1025 m, on bark, 2012.07.06, S.Y. Kondratyuk et al. 121894;

9. Phaeophyscia leana (Tuck.) Essl.

Specimen examined. GANGWON PROVINCE, Wonju City, Socho Town, Mt. Chiak, 37°16'60"N, 128°01'05"E, 465–480 m, on bark, 2004.08.13, J.S. Hur 040554.

10. *Phaeophyscia limbata* (Poelt) Kashiw.

Examined specimens. CHUNGCHEONGBUK PROVINCE. Danyang County, Gagok Town, Mt. Sobaek: 36°57'27"N, 128°26'36"E, 594 m, 2003.10.01, J.S. Hur 030708, 030720; 36°53'22"N, 128°25'54"E, 909 m, on *Quercus*, 2007.06.10, J.S. Hur 070408; 36°55'48"N, 128°27'46"E, 1359 m, on bark, 2007.04.25, J.S. Hur 070340. Goesan County, Yeonpung Town, Mt. Joryeong forest resort, 37°48'27"N, 128°03'32"E, 500 m, on *Quercus*, 2008.07.10, J.S Hur 080307, 080311. Jecheon City, Hansu Town, Mt. Worak, 36°51'37"N, 128°05'27"E, 245 m, on bark, 2004.09.18, J.S Hur 041162; on rock, 2004.09.18, J.S Hur 041161; 2004.09.19, J.S Hur 041225. Yeongdong County, Sangchon Town, Mulhan road, Mt. Samdobong, 36°01'09"N, 127°52'34"E, 1045 m, on *Quercus*, 2015.07.01, J.J. Woo et al. 150457, 150472. CHUNGCHEONGNAM PROVINCE. Gongju City, Banpo Town, Mt. Gyeryong, 36°21'47"N, 127°13'30"E, 440 m, on *Quercus*, 2004.10.23, J.S Hur 041617. GANGWON PROVINCE. Chuncheon City, Buksan Town, Jogyo road, Mt. Maebong, 37°54'40"N, 127°58'58"E, 610 m, on *Quercus*, 2010.05.26, X.Y. Wang et al. 100566, 100569, 100594. Gangneung City: Mt. Seok-Byeong, 37°34'40"N, 128°51'43"E, 0 m, on bark, 2015.07.10, J. S. Park, 151791-3; Wangsan Town, 37°35'23"N, 128°48'42"E, 876 m, on bark, 2014.07.16, U. Jayalal et al. 141370. Hongcheon County, Nae Town, Mt. Eungbok, Tongbaram Valley, 37°51'22"N, 128°30'59"E, 1192 m, on rock, 2009.05.23, Y. Joshi et al., 090667, 090672, 090730. Jeongseong County: tourist pass toward peak Seokbyeongsan: 37°34'39"N, 128°51'24"E, 760 m, on bark, 2015.07.10, S.Y. Kondratyuk & L. Lőkös 150955, 150956, 150957; 37°34'42"N, 128°51'38"E, 810 m, on bark, 2015.07.10, S.Y. Kondratyuk & L. Lőkös 151005; 37°34'37"N, 128°51'47"E, 840 m, on bark, 2015.07.10, S.Y. Kondratyuk & L.Lőkös 151051, 151091, 151160, 151221; Bukpyeong Town, Mt. Baekseokbong, 37°28'44"N, 128°39'46"E, 494 m, on rock, 2009.05.16, Y. Joshi et al. 090425; Jeongseon Village, Mt. Gariwang, 37°25'01"N, 128°32'47"E, 500 m, on *Quercus*, 2008.05.10, J.S Hur 080004. Pyeongchang County: Daegwanryeong Town, Mt. Hwangbyeong, 37°44'44"N, 128°37'30"E, 779 m, on *Quercus*, 2008.07.14, J.S Hur 080371, 080458, 080469, 080371, 080383; Jinbu Town: Mt. Duta (Mt. Bakji), 37°33.950'N, 128°33.161'E, 769 m, on rock, 2010.05.27, X.Y. Wang et al. 100717; Mt. Odae:

37°45'48"N, 128°36'40"E, 998 m, on *Quercus*, 2008.07.15, J.S. Hur, 080485, 080526, 080555. Sacheon Town, Gadunji road, small pine plantation along the seashore road (Haean-ro) at Sacheon Beach, 37°49'43"N, 128°52'42"E, 5 m, on bark, 2015.07.09, S.Y. Kondratyuk & L. Lőkös 150836. Samcheok City: Hajang Town, Mt. Sambong: 37°18'18"N, 128°56'22"E, 930 m, on *Quercus*, 2009.05.15, Y. Joshi et al. 090348; 37°18'18"N, 128°56'22"E, 930 m, on *Quercus*, 2009.05.15, Y. Joshi et al. 090358; Miro Town, Mt. Duta, 37°26'21"N, 128°59'03"E, 955 m, on *Quercus*, 2008.05.11, J.S Hur 080134-2, 080138; Singi Town, Macha road, Mt. Gitdaebong, 37°18'20"N, 128°56'22"E, 1222 m, on *Quercus*, 2009.05.15, Y. Joshi et al. 090393, 090415; Wondeok Village, Mt. Keumbong, 37°14'09"N, 129°17'22"E, 0 m, on bark, 2015.07.11, J.S. Park, 151858-1. Sokcho City, Mt. Seorak, 38°09'59"N, 128°27'16"E, 463 m, on bark, 2009.05.24, Y. Joshi et al. 090822. Taebaek City: Gumunso, Mt. Hambaek, 37°11'58"N, 128°54'56"E, 1268 m, on bark, 2003.08.22, J.S Hur 030682, 030389; Sodo, Mt. Taebaek: 1210 m, on *Betula*, 2004.09.12, J.S Hur, 041037; 1215 m, on *Quercus*, 2004.09.12, J.S Hur 041040, 041041; 37°05'10"N, 128°58'17"E, 0 m, on *Quercus*, 2007.06.18, J.S Hur, 070542; 37°12'52"N, 128°55'38"E, 1323 m, on *Quercus*, 2008.05.25, J.S Hur, 080292; 37°05'07"N, 128°57'29"E, on *Quercus*, 2007.06.18, J.S Hur 070550. Yangyang County, Seo Town, Galjeongokbong: 37°52'48"N, 128°31'02.4"E, 1020 m, on *Quercus*, 2009.05.22, Y. Joshi et al. 090531, 090887; 38°05'26"N, 128°24'39"E, 750 m, on *Carpinus*, *Acer*, 2009.05.25, Y. Joshi et al. 090901, 090904, 090940. Yeongwol County, Sangdong Village: Gurae road, nearby Manhang-jae, 37°09'21"N, 128°53'28"E, 1260 m, on bark, 2014.07.17, U. Jayalal et al. 141282, 141308, 141337; Mt. Jang, 880 m, on rock, 2010.05.29, X.Y. Wang et al. 100899. GYEONGGI PROVINCE, Pocheon City, Soheul Village, Korea National Arboretum: 37°45.550'N, 127°10.253'E, 114 m, on *Juniperus*, 2010.09.15, X.Y. Wang et al. 100950, 100965; 37°45'16"N, 127°09'52"E, 137 m, on bark, 2014.07.10, U. Jayalal et al. 141206, 141210, 141213; Jikdong road, surrounding mountains of National Arboretum, 37°44'57"N, 127°09'05"E, 242 m, on bark, 2014.07.09, U. Jayalal et al. 141172, 141194, 141199, 141204. Yangju City, Nam Town, Cheong road, 38°6'11"N, 128°5'41"E, 750 m, on bark, 2014.07.15, U. Jayalal et al. 141387. GYEONGSANGBUK PROVINCE. Bonghwa County, Mulya Town, Mt. Seondal, 37°01'06"N, 128°43'03"E, 520 m, on bark, 2007.04.24, J.S Hur 070220. Gimcheon City, Daehang Town, Unsu road, Mt. Hwanghak, 36°06'16"N, 127°58'00"E, 828 m, on *Quercus*, 2015.07.02, J.J. Woo et al. 150507, 150513. Yeongju City, Buseok Town, Bukji road, Buseoksa Temple, 36°57'32"N, 128°29'17"E, 1364 m, on rock, 2003.10.04, J.S. Hur 030834. GYEONGSANGNAM PROVINCE. Geochang County, Wicheon Town, Mt. Geumwon, 35°43'40"N, 127°45'50"E, 1351 m, on bark, 2010.06.25, X.Y. Wang et al. 100552; 1153 m, on *Quercus*, 2010.06.25, X.Y. Wang et al. 100505. Hamyang County, Seosang Town, Mt. Baekun, 35°36'20"N, 127°39'20"E, 917–1100 m, on *Quercus*, 2010.06.24, X.Y. Wang et al. 100380, 100395, 100400, 100447. Hapcheon County, Gaya Town, Mt. Gayasan,, 37°48'45"N, 128°06'57"E, on bark, 2004.04.15, J.S. Hur 040202. Sancheong County, Sancheong Village, Mt. Ungseok, 35°22'52"N, 127°51'00"E, on *Quercus*, 2007.10.16, J.S Hur 070852, 070858, 070859; 173 m, on cherry, 2010.10.11, X.Y. Wang & J.A. Ryu, 101344. Incheon Metropolitan City, Ganghwa County, Samsan Town, Seokmo island, 37°44'18"N, 126°19'16"E, 78 m, on *Zelkova*, 2010.09.29, 101022. JEJU PROVINCE. 33°23'18"N, 126°29'45"E, 975 m, on bark, 2004.08.27, J.S Hur 040692, 040699, 040732; 33°27'15"N, 126°33'41"E, 370 m, on bark, 2004.08.29, J.S Hur 040860. Jeju City: Aewol Village, Nabeup road, subtropical forest, 33°26'05"N, 126°19'49"E, 106 m, on rock, 2012.07.05, S.O. Oh et al. 121494, 121833; Nohyeong, Temple Cheonwang, 33°24'39.4"N, 126°29'38.05"E, 681 m, on bark, 2014.06.20, S.Y. Kondratiuk 140670-1, 140670-2. JEOLLABUK PROVINCE. Jangsu County, Jangsu Village, Daeseong road, Mt. Palgongsan: 35°36'01"N, 127°27'57"E, 688 m, on bark, 2005.09.10, J.S Hur 050395, 050411, 050404, 050408; 35°42'32"N, 127°39'36"E, 873 m, on *Quercus*, 2015.07.03, J.J. Woo et al. 150646, 150659, 150605. Muju County: Mupung Town, Mt. Daedeok: 35°54'36"N, 127°52'57"E, 981 m, on *Quercus*, 2015.06.19, J.J. Woo et al. 150116; 35°48'41"N, 127°43'59"E, 1382 m, on bark, 2006.08.10, J.S Hur 060545; Seolcheon Town, Mt. Sambong, 35°52'20"N, 127°49'36"E, 939–970 m, on *Quercus*, 2015.06.18, J.J. Woo et al. 150002, 150019, 150022, 150035, 150037, 150046. Namwon City: Ayeong Town, Gusang road, Mt. Bonghwa, 35°31'52"N, 127°34'05"E, 685 m, on *Castanea*, 2015.07.17, J.J. Woo & S.O. Oh 152192, 152193, 152223; Sandong-myenon & Unbong Village, Mt. Gonam, 35°28'38"N, 127°30'23"E, 685 m, on *Zelkova*, 2015.07.18, J.J. Woo & S.O. Oh 152264. JEOLLANAM PROVINCE. Gurye County: Gwangui Town, Hanguktongsin road, in the park of Sunchon University Education Centre, 34°14'56"N, 127°28'24"E,

90 m, on bark, 2015.06.26, S.Y. Kondratyuk et al. 150431; Toji Town, Mt. Jiri, 35°16'07"N, 127°34'30"E, 504 m, on bark, 2006.09.27, J.S Hur 060751. Gwangyang City, Okryong Town, Mt. Baekun, 35°06'23"N, 127°36'15"E, 664 m, on bark, 2006.06.27, J.S Hur 060383. Haenam County, Songgi Town, Mt. Dalma, 34°22'46"N, 126°34'43"E, 265 m, on bark, 2005.07.26, J.S Hur 050323. Suncheon City: along the Dongcheon river, 34°57'46"N, 127°29'17"E, on cherry, 2011.10.02, S.Y. Kondratyuk et al. 110978; Songgwang Town, Mt. Jogye, 34°59'39"N, 127°20'00"E, 245 m, on rock, 2004.01.31, J.S Hur 040014. Wando County: Bogil Town, Bogil island, on route from Buyong road to Mt. Gyeokja, 34°08'N, 126°32'E, 2 m, on bark, 2010.02.05, Y. Joshi et al. 100002; Gunoe Town, Wando Arboretum, 34°21'10"N, 126°41'10"E, 535 m, on rock, 2005.04.13, J.S Hur 050151. Yeongam County, Haksan Town, Hakgye road, Mt. Heukseoksan, 34°41'21"N, 126°40'51"E, 230 m, on bark, 2005.09.23, J.S Hur 050485, 050496.

11. Phaeophyscia primaria (Poelt) Trass

Examined specimens. CHUNGCHEONGBUK PROVINCE. Danyang County, Gagok Town, Mt. Sobaek: 36°54'04"N, 128°26'29"E, 874 m, on moss over rock, 2007.04.25, J.S. Hur 070310; 36°55'31"N, 128°26'50"E, 1280 m, on moss over rock, 2007.04.25, J.S. Hur 070329; 36°54'49"N, 128°27'33"E, 726 m, on moss over rock, 2007.04.25, J.S. Hur 070375; 36°32'41"N, 127°51'19"E, 440 m, on moss over rock, 2006.04.21, J.S. Hur 060026; Jecheon City, Hansu Town, Mt. Worak, 36°51'36"N, 128°05'27"E, 245 m, on moss over rock, 2004.09.18, J.S. Hur 041160, 041164. GANGWON PROVINCE. Inje County, Buk Town: Baekdam Temple, 38°11'16"N, 128°21'42"E, 450 m, on moss over rock, 2004.10.11, J.S. Hur 041507, 041524; Yongdae road, Mt.Seolak (Beakdamsa course), 275 m, on moss over rock, 2013.03.31, U. Jayalal & J. S. Park 130019; Jeongseon County, Bukpyeong Town, Mt. Baekseokbong, 37°28.739'N, 128°39.760'E, 494 m, on moss over rock, 2009.05.16, Y. Joshi et al. 090429, 090430, 090437, 090441, 090471, 090478, 090495, 090497, 090503, 090505, 090508; Pyeongchang County, Jinbu Town, Suhang road, Mt. Duta(Mt. Bakji), 37°33.943'N, 128°35.025'E, 355 m, on moss over rock, 2010.05.27, X.Y. Wang et al. 100696; Taebaek City, Sodo, Mt. Taebaek, 37°05'10"N, 128°58'17"E, 840 m, on moss over bark, 2007.06.18, J.S. Hur 070536; 36°44'42"N, 128°15'54"E, 1062 m, on moss over bark, 2007.06.18, J.S. Hur 070831; Yangyang County, Seo Town, Hwangi road, Micheongol valley, 37°56.106'N, 128°31.877'E, 420 m, on moss over rock, 2009.05.14, Y. Joshi et al. 090325. GYEONGGI PROVINCE. Pocheon City, Idong Town, 38°06'24.8"N, 127°23'20.5"E, 535 m, on moss over rock, 2008.07.28, J.S. Hur 080338; GYEONGSANGBUK PROVINCE. Bonghwa County, Myeongho Town, Mt. Cheongryang, Cheongryang Temple, 36°47'21"N, 128°54'49"E, 490 m, on moss over rock, 2004.02.28, J.S. Hur 040088. Mungyeong City, Mungyeong Village, Mungyeongsaejae Provincial Park, 36°47'21.3"N, 128°04'26.3"E, 468 m, on moss over soil, 2017.01.01, D. Liu 170619, 170624; Uljin County, Seo Town, Bulyeong Temple, 180 m, on moss over rock, 2003.04.22, J.S. Hur 030142, 030143. GYEONGSANGNAM PROVINCE. Geochang County, Wicheon Town, Mt. Geumwon, 35°43.390'N, 127°47.314'E, 704 m, on moss over rock, 2010.06.25, X.Y. Wang et al. 100560. Hadong County, Hwagae Town, Mt. Jiri, 35°19.679'N, 127°39.522'E, 1346 m, on moss over rock, 2009.10.15, Y. Joshi et al. 091298. Hamyang County: Macheon Town, Chang-won road, Mt. Baekun, 35°37'30"N, 127°37'43"E, 1149 m, on moss over trunk, 2015.07.04, J.J. Woo et al. 150785; Seosang Town, Mt. Baekun, 35°36'16"N, 127°38'26.9"E, 893 m, on moss over rock, 2006.08.17, J.S. Hur 060582. Hapcheon County, Gaya Town, Mt. Gayasan, 35°48'11.9"N, 128°08'35.0"E, 500m, on rock, J.S. Hur, 060107. Sancheong County, Sicheon Town, Mt. Jiri, Jungsan road, 35°18'40"N, 127°44'36"E, 836 m, on moss over rock, 2006.09.15, J.S. Hur 060632. JEJU PROVINCE. Seogwipo City, Seongsan Village, Goseong road, Seopjicoji, 33°19'21"N, 126°50'49"E, 69 m, on moss over rock, 2014.06.19, S.Y. Kondratiuk 140328. JEOLLABUK PROVINCE. Jeongeup City, Naejang, Mt. Naejang, 35°48'11"N, 129°18'47"E, 600 m, on moss over rock, 2003.06.29, J.S. Hur 030450; 690 m, on moss over rock, 2003.06.29, J.S. Hur 030467; 35°29'44.2"N, 126°53'44.5"E, 583 m, on moss over rock, 2003.08.08, J.S. Hur 030605; 35°29'44"N, 126°53'41"E, 535 m, on moss over rock, 2005.01.08, J.S. Hur 050006; Jinan County, Bugwi Town, Suhang road, Mt. Bugwisan, 35°48'20"N, 127°23'40"E, 810 m, on moss over rock, 2005.09.11, J.S. Hur 050439, 050444; Mt. Jiri, on rock, 200303.22, J.S. Hur 030045.

12. *Phaeophyscia pyrrhophora* (Poelt) D.D. Awasthi & M. Joshi

Examined specimens. CHUNGCHEONGBUK PROVINCE. Danyang County, Gagok Town, Mt. Sobaek, 36°55'31''N, 128°26'43''E, on bark, 2007.04.25, J.S. Hur 070325; Yeongdong County, Sangchon Town, Mulhan road, Mt. Samdobong: 36°01'10''N, 127°52'33''E, 1099 m, on trunk, 2015.07.01, J.J. Woo et al. 150465; 36°01'13''N, 127°52'34''E, 1111 m, on trunk, 2015.07.01, J.J. Woo et al. 150480. GANGWON PROVINCE. Pyeongchang County, Daegwanryeong Town, Mt. Hwangbyeong, 37°44'41''N, 128°37'31''E, 630 m, on bark, 2008.07.14, J.S. Hur 080350. Hoengseong County, Dunnae Town, Cheongtaesan, 37°30'40''N, 128°18'04''E, 1200 m, on bark, 2013.06.15, S.O. Oh & J.S. Park 130536. Pyeongchang County, Jinbu Town, Mt. Odae, 37°46'17.2''N, 128°36'04.1''E, 1454 m, on bark, 2008.07.15, J.S. Hur 080544. Taebaek City, Gumunso, Mt. Hambaek: 37°11'12''N, 128°54'56''E, 1361 m, on bark, 2007.06.19, J.S Hur 070703; 37°09'40''N, 128°55'10''E, 1482m m, on rock, 2007.06.19, J.S. Hur 070768; Sodo: 37°05'52''N, 128°57'05''E, 1362 m, on bark, 2014.12.23, S.O. Oh et al. 141541; Mt. Taebaek, 1340 m, on rock, 2004.09.12, J.S. Hur 041083; on bark, 2004.09.12, J.S. Hur 041119; 37°56'48''N, 128°28'03''E, 1225 m, on wood, 2003.11.02, J.S. Hur 030867; 37°05'31.3''N, 128°56'34.2''E, 1521m, on bark, 2007.06.18, J.S. Hur 070613. Yeongwol County, Sangdong Village, Gurae road, nearby Manhang-jae, 37°9'21''N, 128°53'28''E, 1260 m, on bark, 2014.07.17, U. Jayalal et al. 141289. GYEONGSANGBUK PROVINCE. Bonghwa County, Mulya Town, Mt. Seondal, 37°02'13''N, 128°42'42''E, 1238 m, on bark, 2007.04.24, J.S. Hur 070263, 070268. Yeongju City, Buseok Town, Bukji road, Buseoksa Temple, 36°57'32''N, 128°29'17''E, 1364 m, on rock, 2006.08.11, 060561. GYEONGSANGNAM PROVINCE. Hamyang County, Macheon Town: Chang-won road, Mt. Baekun, 35°38'30''N, 127°37'13''E, 1071 m, on trunk, 2015.07.04, J.J. Woo et al. 150731, 150733, 150751, 150759; Mt. Jiri, 935 m, on bark, 2004.09.04, J.S. Hur 040978; Mt. Baekun, 35°37.314'N, 127°38.922'E, 1108 m, on bark, 2010.06.24, X.Y. Wang et al. 100460. Hapcheon County, Gaya Town, Mt. Gayasan: 35°47'54''N, 128°05'56''E, 1040 m, on bark, 2004.04.15, 040203; 35°48'53''N, 128°07'31''E, 1170 m, on bark, 2006.05.05, J.S. Hur 060131. Sancheong County, Sicheon Town, Jungsan road, Mt. Jiri: 35°19'50''N, 127°44'00''E, 1700 m, on bark, 2006.09.15, J.S Hur 060693; 35°19'53''N, 127°43'04''E, 1628 m, on bark, 2006.09.16, J.S Hur 060700. JEJU PROVINCE. 33°22'42''N, 126°35'57''E, 965 m, on bark, 2004.08.28, J.S. Hur 040768; 33°23'18''N, 126°29'45''E, 975 m, on bark, 2004.08.27, J.S. Hur 040698. Jeju City, Halla Mt.: 33°21'30''N, 126°30'14''E, 1633 m, on bark, 2014.11.09, S.O Oh et al. 141443, 141444; 1000 m, on bark, 2008.08.09, J.S. Hur 080717; 33°21'19''N, 126°30'20''E, 1630 m, on bark, 2008.08.10, J.S. Hur 080803; 33°22'44''N, 126°35'7''E, 1000 m, on bark, 2009.04.21, X.Y. Wang et al. 090096; Gwaneumsa Trail, 33°23'37''N, 126°32'16''E, 1072 m, on bark, 2012.06.01, S.O. Oh et al. 121067; Seongpanak Trail: 33°22'N, 126°34'E, 1210–1250 m, on bark, 2012.08.07, S.Y. Kondratyuk et al. 121702; 33°22'34''N, 126°34'12''E, 1209 m, on tree, 2012.08.07, S.Y. Kondratyuk et al. 121682; 33°22'38''N, 126°34'16''E, 1181 m, on bark, 2012.08.07, S.Y. Kondratyuk et al. 121660; 33°22'48''N, 126°35'26''E, 1025 m, on bark, 2012.07.06, S.Y. Kondratyuk et al. 121893; Donnaeko Trail, 33°20'31''N, 126°32'56''E, 1221 m, on bark, 2012.06.20, S.O. Oh et al. 121275; Yeongsil Trail: 33°21'22''N, 126°29'48''E, 1533 m, on tree, 2012.07.04, S.Y. Kondratyuk et al. 121335; 35°18'16''N, 127°34'14''E, 1290 m, on bark, 2016.07.04, D. Liu 162409; 33°21'59.2''N, 126°30'10.4''E, 1560 m, on bark, 2009.04.21, J.S Hur et al. 090168. JEOLLABUK PROVINCE. Muju County: Mupung Town, Mt. Daedeok: 35°55'19''N, 127°52'59''E, 1238 m, on trunk, 2015.06.19, J.J. Woo et al. 150189; 35°54'52''N, 127°53'16''E, 1239 m, on trunk, 2015.06.19, J.J. Woo et al. 150180; 35°54'36''N, 127°52'57''E, 981 m, on trunk, 2015.06.19, J.J. Woo et al. 150117; Seolcheon Town, Mt. Deogyu: 35°51'13''N, 127°44'56''E, 1582 m, on bark, 2006.08.10, J.S. Hur 060472, 060482; 35°45'53''N, 127°40'52''E, 1499 m, on bark, 2006.08.11, J.S. Hur 060573; 35°51'24''N, 127°44'53''E, 1580 m, on bark, 2005.04.03, 050092; 35°51'13''N, 127°44'56''E, 1582 m, on bark, 2006.08.10, J.S Hur 060474; 35°51'10''N, 127°44'56''E, 1571 m, on bark, 2006.08.10, J.S. Hur 060489; Seolcheon Town, Mt. Sambong, 35°52'24''N, 127°50'42''E, 1138 m, on trunk, 2015.06.18, S.O Oh et al. 150084, 150085. JEOLLANAM PROVINCE. Gurye County, Mt. Jiri, 35°17'37''N, 127°32'58''E, 1410 m, on bark, 2006.06.17, J.S. Hur 060234, 060235; on bark, 2006.06.17, J.S. Hur 060299; 35°19'07''N, 127°39'47''E, 1470–1515 m, on bark, 2004.04.23, J.S. Hur 040322; 35°18'50''N, 127°36'12''E, 1413 m, on bark, 2006.09.28, J.S Hur 060847. Gwangyang City, Okryong Town, Mt. Baekun, 35°04'09''N, 127°39'24''E, 985 m, on bark, 2004.09.25, J.S. Hur 041290.

13. Phaeophyscia rubropulchra (Degel.) Moberg

Examined specimens. GANGWON PROVINCE. Jeongseong County, Gangneung City, tourist pass toward peak Seokbyeongsan, 37°34'39"N, 128°51'24"E, 760 m, on bark, 2015.07.10, S.Y. Kondratyuk & L. Lőkös 150954. Yeongwol County, Sangdong Village, Gurae road, nearby Manhang-jae, 37°9'21"N, 128°53'28"E, 1260 m, on bark, 2014.07.17, U. Jayalal et al. 141303, 141304, 141329. Pocheon City, Sohol Village, Jikdong road, National arboretum, 37°45'16"N, 127°09'52"E, 137 m, on bark, 2014.07.10, U. Jayalal et al. 141271. GYEONGSANGBUK PROVINCE. Andong City, Dosan Town, Woncheon road, nearby Wangmo mountain fortress wall, 36°43'25"N, 128°52'50"E, 420 m, on bark, 2014.07.28, J.S. Park & J.J. Woo, 141402. GYEONGSANGNAM PROVINCE. Geochang County, Muju County, Seolcheon Town, Mt. Sambong, 35°52'07"N, 127°49'42"E, 970 m, on rock, 2015.06.18, S.O. Oh et al. 150014, 150020. JEJU PROVINCE. Jeju City, Temple Gwanum, 33°25'22"N, 126°33'35"E, 615 m, on rock, 2012.07.07, S.Y. Kondratyuk & L. Lőkös 121921, 121926, 121930. Seogwipo City, Namwon Village, Mt. Halla Yeongsil trail, 33°21'11"N, 126°29'48"E, 1290 m, on rock, 2016.07.04, D. Liu 162431. JEOLLABUK PROVINCE, Muju County, Mupung Town, Mt. Daedeok, 35°54'36"N, 127°52'57"E, 981 m, on rock, 2015.06.19, S.O. Oh et al.150132. Namwon City, Ayeong Town, Gusang road, Mt. Bonghwa, 35°31'49"N, 127°34'06"E, 688 m, on trunk, 2015.07.17, J.J. Woo & S.O. Oh 152181, 152188, 152196; 35°32'38.2"N, 127°34'21.9"E, 811 m, 2015.07.17, J.J. Woo & S.O. Oh 152223. JEOLLANAM PROVINCE. Gwangyang City, 34°56.696'N, 127°40.635'E, 47 m, on bark, 2010.01.16, J.S. Hur & M.H. Jeong, GW1009. Jangheung County, Gwansan Village, Okdang road, Cheongwansan Mts, near the entrance, 34°32'55"N, 126°55'60"E, 91 m, on bark, 2015.06.23, S.Y. Kondratyuk & L. Lőkös 150425. Sinan County, Bigeum Town, Mt. Sunwang, 92 m, on rock, 2013.06.05, S.O. Oh et al.130163. Yeosu City, Hwayang Town, Yongju road, Najin elementary school yard, 34°42'30"N, 127°36'44"E, 15 m, on bark, 2013.07.28, S.Y. Kondratyuk et al.130770.

14. Phaeophyscia sonorae Essl

Specimen examined. GYEONGSANGNAM PROVINCE, Hamyang County, Seosang Town, Mt. Baekun, 35°36.335'N, 127°39.658'E, 917 m, on *Quercus*, 2010.06.24, X.Y. Wang et al. 100376.

15. Phaeophyscia spinellosa Kashiw.

Examined specimens. JEJU PROVINCE. Jeju City: Hallim Village, Gwideok road, coast near the Chorok village, 33°26'33"N, 126°17'00"E, 18 m, on rock, 2012.07.05, S.Y. Kondratyuk et al, 121370; Hangyeong Town, Sinchang road, nearby coast around Singaemul Park, 33°30'31"N, 126°10'13"E, 19 m, on rock, 2012.07.05, S.Y. Kondratyuk et al, 121413, 121415, 121416. Seogwipo City: Daejeong village, Sangmo road, around Mt. Song-ac nearby coast, 33°12'35"N, 126°17'30"E, 16 m, on rock, 2012.07.05, S.Y. Kondratyuk et al, 121427; Seongsan Village, Goseong road, Seopjicoji, 33°19'21"N, 126°50'49"E, 69 m, on rock, 2014.06.19, S.Y. Kondratiuk 140328, L. Lőkös 140387-3. JEOLLANAM PROVINCE. Goheung County, Yeongnam Town, Ucheon road, Yongam village, Yongbawi seaside, 34°35'41"N, 127°30'19"E, 10 m, on rock, 2010.02.19, Y. Joshi et al. 100337, 100193. Jindo County, Jeob island, 34°23'41"N, 126°18'9"E, 1 m, on rock, 2011.06.03, X.Y. Wang & J.A. Ryu, 110504, 110510, 110514, 110632. Wando County, Cheongsan island, 34°09'10"N, 126°52'50"E, 2 m, on rock, 2011.06.23, X.Y. Wang & J.A. Ryu 110727, 110741.

16. Phaeophyscia squarrosa Kashiw.

Examined specimens. CHUNGCHEONGBUK PROVINCE. Yeongdong County, Sangchon Town, Mulhan road, Mt. Samdobong, 36°01'14"N, 127°52'41"E, 982 m, on trunk, 2015.07.01, J.J. Woo et al. 150435. GANGWON PROVINCE. Chuncheon City, Buksan Town: Jogyo road, Mt. Maebong, 37°54.797'N, 127°59.099'E, 685 m, on *Quercus*, 2010.05.26, X.Y. Wang et al. 100624. Hongcheon County, Nae Town, Mt. Eungbok, 37°51.772'N, 128°33.528'E, 866 m, on *Quercus*, 2009.05.23, Y. Joshi et al. 090745. Jeongseong County, Gangneung City, tourist pass toward peak Seokbyeongsan, 37°34'38.58"N, 128°51'23.94"E, 760 m, on bark, 2015.07.10, S.Y. Kondratyuk 121427, 150956, 151092, 150953; Mt. Baekseokbong, 37°28.739'N, 128°39.760'E, 494 m, on bark, 2009.05.16, Y. Joshi et al. 090435. Pyeongchang County: Daegwanryeong Town, Mt. Hwangbyeong, 37°44'41"N, 128°37'31"E, 630 m,

on bark, 2008.07.14, J.S. Hur 080349, 080351, 080388, 080389,080464; Jinbu Town, Mt. Odae, 37°46'04.2"N, 128°36'12.4"E, 1277 m, on *Quercus*, 2008.07.15, J.S. Hur 080506, 080508, 080522, 080537, 080548, 080594, 080603, 080614; 37°46'06"N, 128°35'38"E, on bark, 2004.05.08, J.S. Hur 040492; Suhang road, Mt. Duta (Mt. Bakji), 37°34.390'N, 128°36.233'E, 989 m, on rock, 2010.05.27, X.Y. Wang et al. 100768. Samcheok City, Miro Town, Mt. Duta, 37°25'54"N, 128°58'33"E, 1352 m, on bark, 2008.05.11, J.S. Hur 080167. Sokcho City, Mt. Seorak: 38°09.981'N, 128°27.267'E, 463 m, on *Quercus*, 2009.05.24, Y. Joshi et al. 090761, 090806; 37°52.796'N, 128°31.040'E, 1020 m, on *Quercus*, 2009.05.22, Y. Joshi et al. 090524. Taebaek City, Sodo, Mt. Taebaek, on bark, 2004.09.12, J.S. Hur 041157-1. Yangyang County, Seo Town, Galjeongokbong, 37°52.919'N, 128°30.188'E, 1084 m, on *Quercus*, 2009.05.22, Y. Joshi et al. 090546, 090565, 090567, 090595, 090647, 090595. GYEONGSANGBUK PROVINCE. Mungyeong City, Sanbuk Town, Mt. Gongdeok, 36°44'42"N, 128°15'54"E, on bark, 2007.06.20, J.S. Hur 070774. GYEONGSANGNAM PROVINCE. Hamyang County, Seosang Town, Mt. Baekun, 35°36'18"N, 127°38'26"E, 903 m, on bark, 2006.08.17, J.S. Hur 060586. JEOLLANAM PROVINCE, Gurye County, Toji Town, Mt. Jiri, 35°19'26"N, 127°36'46"E, 1555 m, on *Quercus*, 2006.09.28, J. S Hur 060864.

17. Physciella melanchra (Hue) Essl.

Examined specimens. GYEONGNAM PROVINCE. Namhae Co., Mangeun Mt., 34°51.072'N, 127°49.600'E, 160 m, on rock, 2011.04.28, X.Y. Wang & J.A. Ryu 110153, 110157, 110121. Tongyeong City, Salyang Island, 34°50.555'N, 128°12.179'E, 25 m, on rock, 2011.04.20, X.Y. Wang et al. 110016; Yokgi Town, Saryang Island, 34°50'08"N, 128°10'51"E, 44 m, on rock, 2007.03.17, J.S. Hur 070010. Bonghwa County, Myeongho Town, Mt. Cheongryang, Cheongryang Temple, 36°47'21"N, 128°54'49"E, 490 m, on rock, 2004.02.28, J.S. Hur 040089. INCHEON METROPOLITAN CITY. Ganghwa County, Samsan Town, Seokmo Island, 37°41.381'N, 126°19.320'E, 160 m, on rock, 2010.09.29, X.Y. Wang et al. 101048. JEJU PROVINCE. Jeju City: 33°28'25"N, 126°29'47"E, 145 m, on rock, 2004.08.26, J.S. Hur 040625; Aewol village, Nabeup road, subtropical forest, 33°26'06"N, 126°19'48"E, 90 m, on rock, 2012.07.05, S.O. Oh et al. 121829, 121833; Chuja island, Chuja Town, Sinyang-1 road, Seashore of Mojini-mongdol, 33°56'44"N, 126°20'03"E, 57 m, on rock, 2014.06.21, S.Y. Kondratyuk & L. Lőkös 140883-3; Nohyeong, Temple Cheonwang, 33°24'39"N, 126°29'38"E, 681 m, on bark, 2014.06.20, S.Y. Kondratyuk 140682-4. Seogwipo City: Namwon village, Wimi road, 33°16'13"N, 126°39'30"E, 10 m, on rock, 2009.05.29, J.S. Hur et al. 091438; Yongduam, 33°30'57"N, 126°30'38"E, 10 m, on rock, 2009.04.20, J.S. Hur et al. 090090. JEOLLABUK PROVINCE. Gochang County, Asan Town, Mt. Seonun: 50 m, on rock, 2003.05.11, J.S. Hur 030226; 60 m, on rock, 2003.05.11, J.S. Hur 030231; Haenam County, Samsan Town, Mt. Duryun, 200 m, on rock, 2003.06.07, J.S. Hur 030312. Jangheung County: Gwansan village, Okdang road, Cheongwansan Mts, near the entrance, 34°32'55"N, 126°55'60"E, 91 m, on bark, 2015.06.23, S.Y. Kondratyuk 150417, 150419. Namwon City, Unbong village, Sandong-myenon, Mt. Gonam, 35°28'36"N, 127°31'15"E, 545 m, on bark, 2015.07.18, J.J Woo & S.O. Oh, 152247. Shinan County: Heuksan island, 34°39.882'N, 125°26.189'E, 5 m, on rock, 2011.06.21, X.Y. Wang & J.A. Ryu 110559; Sinui Town, Hatae-gil seaside, Sinuido, 1 m, on rock, 2013.06.28, S.O. Oh et al.130608. Suncheon City: along the Dongcheon river, 34°57'46"N, 127°29'17"E, on bark, 2011.10.02, S.Y. Kondratyuk 110979; Sunchon National University: 34º58'01"N, 127º28'47"E, 47 m, on *Cerasus* sp., 2017.12.01, D. Liu 171448, 171449, 171450; humanitarian faculty, 34°58'10"N, 127°28'36"E, on bark, 2011.10.04, S.Y. Kondratyuk 110992; in front of library, 34°58'7"N, 127°29'2"E, on bark, 2011.10.04, S.Y. Kondratyuk 110985; 34°58'12"N, 127°28'57"E, 28 m, on stairs, 2017.06.08, D. Liu 170625; Seokhyeon, Hyanglimsa, 34º58'20"N, 127º28'43"E, 46 m, on rock, 2017.10.04, D. Liu, 171426; Songgwang Town, Mt. Jogye, 150 m, on rock, 2003.04.24, S.O. Oh 030167. Wando County: Geumil village, Wolsong road, Geumil island, 34°20'29"N, 127°02'38"E, 1 m, on rock, 2012.04.19, U. Jayalal et al. 120255; Yaksan Town, Deugam road, 34°21'38"N, 126°53'34"E, 5 m, on rock, 2012.04.18, U. Jayalal et al. 120127; Yaksan Town, Deugam road, Joyak island 34°21'38"N, 126°53'34"E, 5 m, on rock, 2012.04.18, U. Jayalal et al. 120127. Yeosu City: Hwayang Town, Yongju road, along road, 34°30'45"N, 127°44'14"E, 20 m, on *Firmiana simplex*, 2013.07.28, S.Y. Kondratyuk 130755,130757; along the road at seacoast, 34°39'5"N, 127°34'46"E, 8 m, on *Camellia japonica*, *Celtis*, *Quercus variabilis*, *Sorbus*

amurensis, 2013.07.28, S.Y. Kondratyuk 130779, 130780, 130782; Nam Town, Yusong road, Geumoh island, Mando beach, 34°31'37''N, 127°46'13''E, 18 m, on rock, 2012.04.27, U. Jayalal et al. 120578.
