# Supplementary material for: Revision of the Lichen Genus Phaeophyscia and Allied Atranorin Absent Taxa (Physciaceae) in South Korea
Source: Microorganisms. 2019 Aug 6;7(8):242. doi: 10.3390/microorganisms7080242 (PMC6723189; doi:10.3390/microorganisms7080242)
Supplement: Supplementary file 1 [file microorganisms-07-00242-s001.zip › microorganisms-516652-table S1.pdf]

Table S1. ITS sequences of family Physciaceae used in this study.

Taxa	Accession no.	Voucher and Herbarium Information
Anaptychia bryorum	EF582777	Söehting 8378 (UPS)
A. isidiata	EF582780	Himelbrant K-04-10 (LECB)
A. palmatula	EF582779	Ahti 58054 (H)
A. runcinata	EF582776	Odelvik & Karlsson 00243
Heterodermia hypoleuca	**MN150489**	**D. Liu 141437 (KoLRI)***
H. japonica	DQ337322	
H. obscurata	DQ337323	15184d
H. vulgaris	HQ650704	AFTOL-ID 320
Hyperphyscia adglutinata	AF540521	
Hy. adglutinata	AF224361	
Hy. adglutinata	AF250795	
Hy. confusa	JN164684	
Hy. confusa	JN164685	
Hy. confusa	JN164686	
Hy. crocata	**MN150490**	**S.Y. Kondratyuk 120413 (KoLRI)***
Phaeophyscia adiastola	KT695334	
P. adiastola	KT695398	BIOUG24047-D10
P. ciliata	AF224354	
P. ciliata	AF224355	
P. constipata	AF224374	Löfgren 12.111998 (UPS)
P. endococcina	EF582753	Moberg 12253 (UPS)
P. endococcinodes	**MN150503**	**S.O. Oh et al. 130163 (KoLRI)***
P. endophoenicea	AF250798	
P. exornatula	**EU670224**	**Hur 060117 (KoLRI)**
P. hirtusa	GU247167	
P. insignis	GU247169	BCN-Lich 17035
P. sp.1	**EU670226**	**Hur 060029 (KoLRI)**
P. limbata	**EU670221**	**Hur 050411 (KoLRI)**
P. orbicularis	AF250799	
P. orbicularis	AF540528	M. Schultz 9808.021
P. primaria	**EU670216**	**Hur 041524 (KoLRI)**
P. primaria	**EU670215**	**Hur 041160 (KoLRI)**
P. primaria	**MN150491**	**D. Liu 170624 (KoLRI)***
P. pyrrhophora	**MN150492**	**D. Liu 162409 (KoLRI)***
P. pyrrhophora	**EU670227**	**Hur 060299 (KoLRI)**
P. rubropulchra	**MN150493**	**U. Jayalal et al. 141271 (KoLRI)***
P. rubropulchra	**MN150494**	**J.S. Park & J.J. Woo 141402 (KoLRI)***
P. rubropulchra	**MN150495**	**D. Liu 162431 (KoLRI)***
P. sciastra	AF224357	Myllys 1996 s.n.
P. sp.2	**MN150496**	**D. Liu et al. 141442 (KoLRI)***
P. squarrosa	**EU670229**	**Hur 060586 (KoLRI)**
P. squarrosa	**EU670233**	**Hur 060864 (KoLRI)**
P. squarrosa	**MN150497**	**S.Y. Kondratyuk 150953 (KoLRI)***
Physcia aipolia	**MN150498**	**D. Liu 160579 (KoLRI)***
Ps. austrostellaris	GU074409	Elix 38829 (CBG)
Ps. dubia	JQ301695	T. Ahti 69359, S. Stenroos & R. Pino (H)

Ps. erumpens	AF540532	R.Welz 182 (B)
Ps. integrata	AF540533	H. Sipman & R. Welz 44890 (B)
Ps. krogiae	AF540534	H. Sipman & R. Welz 44672 (B)
Physciella sp.1	**MN150502**	**D. Liu 162346 (KoLRI)***
Ph. chloantha	AF250797	
Ph. chloantha	KT695368	
Ph. melanchra	**EU670211**	**Hur 040625 (KoLRI)**
Ph. melanchra	**EU266100**	**Hur 040089 (KoLRI)**
Ph. melanchra	**MN150499**	**D. Liu 170625 (KoLRI)***
Ph. melanchra	**MN150500**	**D. Liu 171426 (KoLRI)***
Ph. melanchra	**MN150501**	**D. Liu 171449 (KoLRI)***
Ph. melanchra	AY498666	
Ph. poeltii	KX132940	LC-049 (WSL)
Physconia perisidiosa	AY368141	MAF 9801
Pn. servitii	AY368143	MAF 9800
Pn. subpulverulenta	DQ862489	MAF-Lich 14116
Pn. thorstenii	DQ862494	MAF-Lich 14120
Pn. venusta	DQ862498	MAF-Lich 14124

Specimens from South Korea were bold, asterisk referred to the newly generated sequences in this study. *A. = Anaptychia, Hy. = Hyperphyscia, P. = Phaeophyscia, Ph. = Physciella, Pn. = Physconia, Ps. = Physcia.*
